# Impact of ACE I gene insertion/deletion, A-240T polymorphisms and the renin–angiotensin–aldosterone system on COVID-19 disease

**DOI:** 10.1186/s12985-023-02283-w

**Published:** 2024-01-10

**Authors:** Christian M. Zobel, Hartmut Kuhn, Maximilian Schreiner, Werner Wenzel, Jasper Wendtland, Cengiz Goekeri, Lorenz Scheit, Klaas Oltmanns, Dominic Rauschning, Marica Grossegesse, Natalie Hofmann, Hubert Wirtz, Sebastian Spethmann, BwKrhs-Covid-Research-Group, Ullrich Baumgarten, Tobias Wageloehner, Nino Neumann, Annette Mueller, Rico Mueller, Jan Philip Krueger, Alena Borchert, Felix Weinreich, Franziska Keidel, Maria Koch, Meike Schüßler

**Affiliations:** 1 Department of Internal Medicine, Bundeswehr Hospital Berlin, Scharnhorstrstr. 13, 10115 Berlin, Germany; 2 Department of Respiratory Medicine, University of Leipzig, Leipzig, Germany; 3 Department of Microbiology and Hospital Hygiene, Bundeswehr Hospital Berlin, Berlin, Germany; 4 Department of Internal Medicine, Bundeswehr Hospital Hamburg, Hamburg, Germany; 5 Department of Internal Medicine, Bundeswehr Hospital Westerstede, Westerstede, Germany; 6 Department of Internal Medicine, Bundeswehr Hospital Koblenz, Koblenz, Germany; 7 Department of Infectious Diseases and Respiratory Medicine, Charité – Universitätsmedizin Berlin, Berlin, Germany; 8 Faculty of Medicine, Cyprus International University, Nicosia, Cyprus; 9 Centre for Biological Threats and Special Pathogens, ZBS1, Robert Koch Institute, Highly Pathogenic Viruses, Berlin, Germany; 10 Deutsches Herzzentrum der Charité Berlin, Berlin, Germany; 11 Charité – Universitätsmedizin Berlin, Corporate Member of Freie Universität Berlin and Humboldt Universität Zu Berlin, Berlin, Germany

**Keywords:** COVID-19, SARS-CoV2, ACE-polymorphism, Renin–angiotensin–aldosteron-system (RAAS), Inflammation

## Abstract

**Background:**

The coronavirus disease 2019 (COVID-19) pandemic is driven by severe acute respiratory syndrome coronavirus 2 (SARS-CoV-2) infection, which has led to an enormous burden on patient morbidity and mortality. The renin–angiotensin–aldosterone system (RAAS) plays a significant role in various pulmonary diseases. Since SARS-CoV-2 utilizes the angiotensin-converting enzyme (ACE)2 receptor to exert its virulence and pathogenicity, the RAAS is of particular importance in COVID 19.

**Methods:**

Our preliminary study investigates retrospectively the influence of selected ACE-polymorphisms (I/D location at intron 16 in the B-coding sequence (rs4646994) and A-240T (rs 4291) at the A-promoter) as well as ACE1 and ACE2 serum levels on disease severity and the inflammatory response in inpatients and outpatients with COVID-19.

**Results:**

Our study included 96 outpatients and 88 inpatients (65.9% male, mean age 60 years) with COVID-19 from April to December 2020 in four locations in Germany. Of the hospitalized patients, 88.6% participants were moderately ill (n = 78, 64% male, median age 60 years), and 11.4% participants were severely ill or deceased (n = 10, 90% male, median age 71 years). We found no polymorphism-related difference in disease, in age distribution, time to hospitalization and time of hospitalization for the inpatient group. ACE1 serum levels were significantly increased in the DD compared to the II polymorphism and in the TT compared to the AA polymorphism. There was no significant difference in ACE 1 serum levels l between moderately ill and severely ill patients. However, participants requiring oxygen supplementation had significantly elevated ACE1 levels compared to participants not requiring oxygen, with no difference in ACE2 levels whereas females had significantly higher ACE2 levels.

**Conclusions:**

Although there were no differences in the distribution of ACE polymorphisms in disease severity, we found increased proinflammatory regulation of the RAAS in patients with oxygen demand and increased serum ACE2 levels in women, indicating a possible enhanced anti-inflammatory immune response.

*Clinical trial registration*: PreBiSeCov: German Clinical Trials Register, DRKS-ID: DRKS00021591, Registered on 27th April 2020.

## Introduction

Coronavirus disease 2019 (COVID-19) is triggered by infection with severe acute respiratory syndrome coronavirus 2 (SARS-CoV2) and represents the greatest global health challenge of the twenty-first century to date. First detected in Wuhan in late 2019 [1], it rapidly spread all over the world. Despite the availability of protective vaccines, it remains a pandemic with significant morbidity and mortality, also due to viral mutations. The clinical manifestation is heterogeneous and ranges from asymptomatic to severe respiratory dysfunction (acute respiratory distress syndrome; ARDS), often accompanied by multiorgan failure [1]. To date, it is not known how the highly heterogeneous disease severity occurs in patients with similar preconditions. The renin–angiotensin–aldosterone system (RAAS) plays a central role in COVID-19 pathogenesis as the spike protein domain of SARS-CoV-2 binds to the cell membrane-anchored metallopeptidase angiotensin-converting enzyme 2 (ACE2) [2]. ACE2 is both, a soluble enzyme and a membrane-bound receptor. The receptor is expressed ubiquitously across various tissues, with abundant expression reported in the lungs and intestines [3]. Furthermore, ACE2 tissue distribution is associated with the clinical pattern of diffuse alveolar damage, pulmonary consolidation, gastroenteritis and systemic vasculitis [4–6]. Increased ACE2 receptor expression on membranes has been reported in males, in the population with an age above 60, patients with metabolic disorders like diabetes and hypertension and patients with respiratory or cardiac diseases [7]. Interestingly, these conditions are also risk factors for a severe course of COVID-19 [8, 9].

The cellular serine protease transmembrane protease serine subtype 2 (TMPRSS2) cleaves the viral spike protein and promotes the fusing of membranes between the cell and the virus [2]. After internalization of the virus and subsequent intracellular replication, the cell membrane bound ACE2 activity is down-regulated at the cell surface [10]. Since SARS-CoV-2 depends primarily on ACE2 for fusion and entry, ACE2 variation is thought to be one of the causes of differences in disease severity.

Furthermore, ACE2 degrades 10-amino-acid-Angiotensin-I (Ang-I) to 9-amino-acid-Angiotensin (Ang1-9), which in turn can be reduced by the dipeptidyl carboxypeptidase Angiotensin-converting enzyme 1 (ACE1) to the bioactive 7 amino-acid-Angiotensin (Ang1-7). Conversely, ACE2 forms the bioactive Ang1-7 directly from the 8-amino-acid Angiotensin II (Ang-II). In addition, Ang-II can also be produced from Ang-I by ACE1 [5, 11, 12].

Ang-II-binding to the Angiotensin-1-Receptor (AT1R) has vasoconstrictive, pro-inflammatory, pro-coagulant and pro-apoptotic effects and leads to oxidative stress, whereas binding to the angiotensin-2 receptor (AT2R) has the opposite effects [13]. Ang1-7 binds to the G-protein-coupled Mas receptor and thereby attenuates the effects of Ang-II through vasodilative, anti-inflammatory, anti-coagulant and anti-apoptotic activities [11, 12, 14]. A dysbalanced RAAS plays a prominent role in the pathogenesis of many lung diseases. Several pulmonary diseases and their activity are associated with elevated levels of ACE1, including sarcoidosis, hypoxic pulmonary hypertension, idiopathic pulmonary fibrosis and ARDS [15–17].

The ACE1 gene is located on chromosome 17q35,131 (rs4646994). The I/D polymorphism of intron 16 induces alternative splicing in the ACE protein [18]. The DD polymorphism is associated with the highest ACE levels in most ethnic groups and the I/D polymorphism shows the greatest variation (47%) in ACE levels [12, 19]. Verma et al. investigated the influence of ACE1 I/D genotype on disease severity in patients with COVID-19 in a northern Indian cohort and showed that the D allele is more prevalent in severe COVID-19 disease [18]. In addition, Stawisk et al. demonstrated differences in COVID-19 susceptibility attributable to numerous genetic polymorphisms [20]. In contrast, Delanghe et al. demonstrated no association of ACE I/D variants in European, Middle Eastern, and North African study cohorts in mortality rate and disease severity [21]. The single-nucleotide polymorphism A240T (rs 4291) is associated with the susceptibility to diseases like arterial hypertension, a dysregulation of glucose and lipid metabolism, higher concentrations of urea and might influence serum ACE levels [22–25].

Nevertheless, different studies showed that neither disease severity of COVID-19 nor sex influenced ACE2 levels [26]. Therefore, we aimed to retrospectively examine ACE1 and ACE2 polymorphisms in outpatient and inpatient cohorts, their role within the RAAS, and the resulting impact on the inflammatory immune response triggered by SARS-CoV-2.

## Materials and methods

### Recruitment, study participants

The present study is a post-hoc analysis of a previously published prospective observational study with hospitalized COVID-19 patients (Frontiers in Microbiology: “Serum interleukin-6, procalcitonin, and C-reactive protein at hospital admission can identify patients at low risk for severe COVID-19 progression” [27]). We have added a second cohort of ambulatory mild affected COVID-19 participants (outpatients). For the latter cohort, anonymized blood cakes from serum tubes were randomly collected in the microbiology laboratory of the Bundeswehr hospital Berlin. These samples were obtained from confirmed COVID-19 infected staff and soldiers of the Bundeswehrkrankenhaus Berlin, soldiers of surrounding military sites and citizens from the urban area of Berlin which did not require inpatient therapy to determine COVID-19 antibodies after the disease. No further information on previous illnesses or even clinical parameters and laboratory results are available for this cohort.

The group of hospitalized COVID-19 patients from our previously conducted study were classified according to the WHO Ordinal Clinical Improvement Scale [28]. Thus, we were able to compare three different groups: (1) Ambulatory mild diseases; (2) Hospitalized moderate diseases: with or without oxygen therapy (WHO score 4,5); (3) Hospitalized severe diseases: Non-invasive ventilation, invasive ventilation, ECMO-therapy or dead (WHO score 6–10). A representative selection of patients with different WHO grades is listed in Table 1. Furthermore, we subdivided the hospitalized with regard to their oxygen demand. The patients from all groups were recruited between April and December 2020.

**Table 1:** Excerpt of patient characteristics that led to the COVID-19 WHO scale

	Age	Sex	BMI	Initial spO2 (O_2_^−^)	Initial spO2 (O_2_^+^)	RR (Sys)	RR (dias)	MAD	High-Flow in the course?	Intubation in the course?	HF (min)	AF (min)	Days of hospitalization
1	53	w	24.2	94	0	105	66	79	No	No	70	14	8
2	79	m	33,59	86	94 (2 l/min)	107	64	78	Yes	Yes	98	16	3
3	78	m	36,7	88	91 (2 l/min)	100	90	93	Yes	Yes	68	20	2
4	75	w	30,4	85	90 (2 l/min)	120	80	93	No	No	84	17	4
5	52	m	38,5	92	95 /2 l/min)	130	76	94	No	No	80	17	8
6	63	m	//	88	96 (2 l/min)	130	82	98	No	No	81	17	10
7	59	m	34,14	//	91 (3 l/min)	147	95	112	Yes	Yes	97	14	16

	Days until hospotalization	Arterial hypertension	ACE- or AT1-blocker	Cardiovascular diseases	Diabetes mellitus	Active tumor disease	Pulmonary diseases	Liver diseases	Chronic kidney diseases	Further disease	COVID-19	COVID-19
											WHO-score	WHO-scale
1	9	No	No	No	No	No	No	No	No	Yes	4	Hospitalised moderate
2	8	No	Yes	Yes	Yes	No	Yes	No	Yes	Yes	8	Hospitalised severe
3	4	Yes	Yes	Yes	No	No	Yes	No	No	Yes	8	Hospitalised severe
4	14	Yes	Yes	No	Yes	No	No	No	No	Yes	5	Hospitalised moderate
5	5	No	No	No	Yes	No	No	No	No	No	5	Hospitalised moderate
6	6	No	No	No	No	No	No	No	No	No	5	Hospitalised moderate
7	10	Yes	Yes	Yes	Yes	No	No	No	No	Yes	8	Hospitalised severe

For the group of inpatients, sera, clinical data, laboratory standard parameters and remaining blood cakes from the serum tubes obtained from hospitalized patients with COVID-19 were provided by the PreBiSeCov study, carried out by the Bundeswehr hospital Berlin [27]. In this study, immunocompetent adults who required inpatient therapy due to COVID-19 were enrolled at the Bundeswehr hospital Berlin, Hamburg, Westerstede and Koblenz. Serum samples, standardized clinical laboratory evaluation and clinical data were collected at various time points during hospitalization, the first within the first 24 h after admission. At the time of the first blood sampling, all participants were acutely infected with COVID-19. Sera were stored at − 20 °C. Hospitalized but not ventilated patients were defined as moderately ill, whereas ventilated were classified as severely affected. ID and AT polymorphisms were analyzed in all remaining blood cakes. ACE1 and ACE2 levels were measured in all serum samples of the PreBiSeCov study.

### SARS-CoV-2 diagnostics

Nasopharyngeal swabs were taken from all participants and a SARS-CoV-2 RT-PCR with invitro diagnostic kits was performed according to manufacturer’s protocol. Due to the early start of the prospective PreBiSeCov study and the fact that PCR diagnostics were not available everywhere at short notice, inpatients with an urgent suspicion of COVID-19 pneumonia or based on computed tomography (CT) morphology were also included in PreBiSeCov study. According to the protocol, a retrospective exclusion was performed if the PCR was negative for COVID-19 twice in the clinical course. In the present study, all serum samples, including ambulatory patients, were analyzed for the presence of neutralizing antibodies to SARS-CoV-2 by the plaque reduction neutralization test (PRNT) [26].The PRNT was performed as previously described [26], except that only a single sample dilution was analyzed instead of a dilution series. Briefly, 100 µL of serum was diluted 1:10 in DMEM (10% FCS, 2 mM l-glutamine), mixed 1:1 with SARS-CoV-2 (strain BetaCoV/Germany/BavPat1/2020, Institute for Microbiology of the German Armed Forces; final virus concentration 1000 TCID50/mL), and incubated for 1 h at room temperature (RT). Subsequently, 100 µL of the diluted serum-virus mixture was added to wells containing 2 × 10^4^ Vero E6 cells per well (#85020206, European Collection of Authenticated Cell Cultures (ECACC)) in a 96-well plate. Each serum sample was run in eight replicates, and cells were incubated at 37 °C and 5% CO_2_ for 5 days. After five days, each well was examined for visible CPE by light microscopy. Samples with at least one neutralized replicate were considered positive. For quality control, a positive control (patient serum with defined titer) was analyzed in parallel and back titration of the virus stock was performed. In the inpatients, the results of the neutralisation assay were the same as those of the PCR. In the outpatient cohort, the neutralization assay confirmed that infection had occurred.

### Genotyping of I/D and A/T polymorphism

The genotyping of ACE polymorphisms was performed with PCR using patient DNA. Genomic DNA was isolated from the blood cakes of the serum tubes with the DNA Mini Kit (Qiagen) according to manufacturer’s instructions. PCR primers used are displayed in Table 2.

**Table 2:** Primers used in PCR reactions

Name	Primersequence 5′ → 3′	Annealing (°C)	PCR-product (bp)
ACE-1 (F) ACE-2 (R)	GCCACTGCTGGAGACCACT AGCTCCAGCCCTTAGCTCAC	61	439
ACE-3 (F) ACE-4 (R)	TGGGACCACAGGCGCCCGCCACTA TCGCCAGCCCTCCCATGCCCATAA	67	336
A240T-1 (F) A240T-2 (R)	GCCCCGGCCTTGTCACTCCGGAGG CCGCAGAGGAAGCTGGAGAAA	63	193

For determination of I/D polymorphisms two different PCR reactions were utilized. Briefly, the first PCR reaction was carried out in a final volume of 20 µL using primer ACE1 and ACE2 with 5 µL DNA (50 ng), 10 pmol of each oligonucleotide primer and 10 µl iQ SYBR Green Supermix (BIO-RAD). The PCR was performed for 42 cycles with an initial denaturation at 95 °C for 5 min, cycling times of 10 s at 95 °C, 30 s at 61 °C, 30 s at 72 °C and a final extension period at 72 °C for 7 min using the CFX Connect Real Time System (BIO-RAD). After amplification was completed, a melting curve analysis was performed by cooling the reaction to 65 °C and then heating slowly to 95 °C. Melting curve and fluorescent signals were analyzed with CFX Manager 3.1 software (Bio-RAD).

**Fig. 1:**
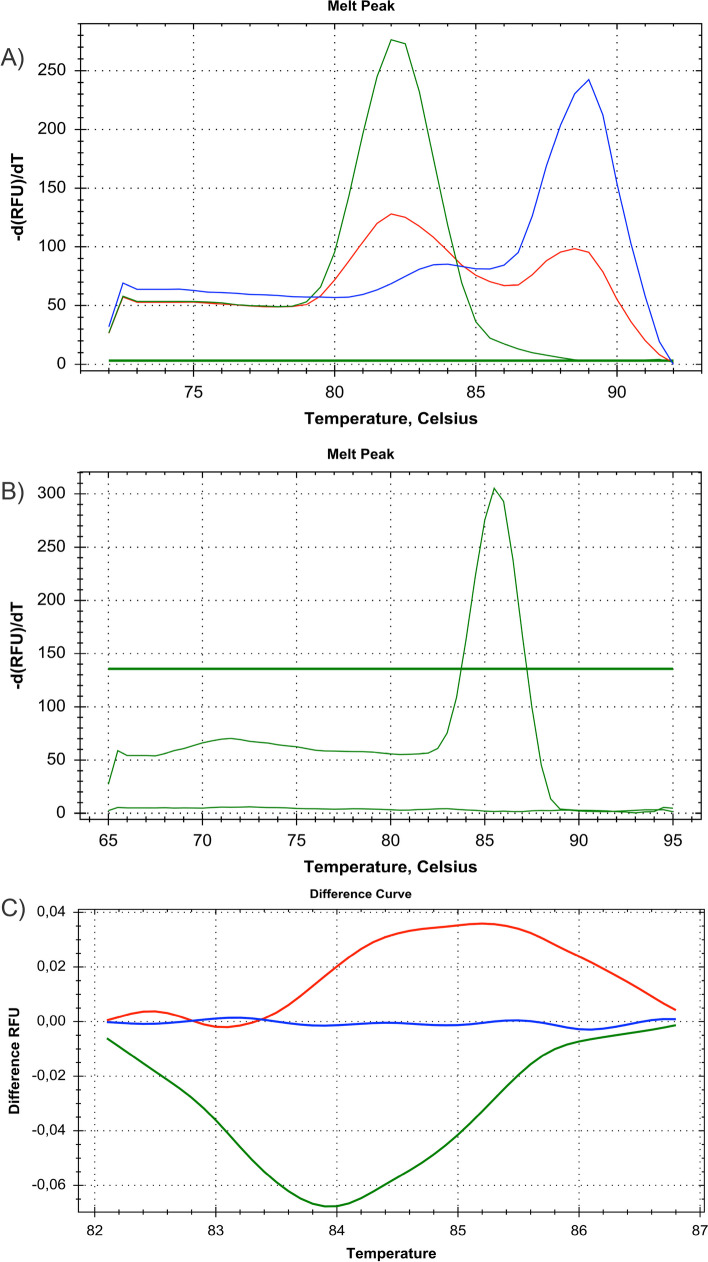
Analysis of Angiotensin-converting enzyme (ACE) polymorphisms. **A** Melting curve analysis of polymerase chain reaction (PCR) with primers ACE1/2 (green line D/D; redline = 1/D; blue line I/I). **B** Melting curve analysis of PCR with primers ACE3/4 (peak = I-positive). **C** HRM analysis of PCR with primers A240T-1/2 (green line [cluster 1] = A/A; blue line [cluster 2] = T/T; red line [cluster 3] = A/T)

As shown in Fig. 1a we found one melting peak for D at 81 °C, one melting peak for I at 88,5 °C and all both peaks for I/D. Due to the weak peaks detected for I at 88.5 °C in I/D, we performed a second PCR using primers ACE3 and ACE4 with equal amounts of constituents as in the first PCR reaction. This second PCR was performed for 32 cycles with an initial denaturation at 95 °C for 5 min, cycling times of 10 s at 95 °C, 30 s at 67 °C, 30 s at 72 °C and a final extension period at 72 °C for 7 min. After melting curve generation analysis of fluorescent signals showed no signal for D and one peak for I at 85.5 °C (Fig. 1b).

For determination of A/T polymorphism, we used one PCR which was carried out in a final volume of 20 µL using primer A240T-1 and A240T-2 with 5 µL DNA (50 ng), 10 pmol of each oligonucleotide primer and 10 µl Precision Melt Supermix (BIO-RAD). This PCR was performed for 40 cycles with an initial denaturation at 95 °C for 2 min and cycling times of 10 s at 95 °C, 30 s at 53 °C, and 30 s at 72 °C and by a final extension period at 72 °C for 7minuites in the CFX Connect Real Time System (BIO-RAD). After melting curve generation, fluorescent signals were analyzed with Precision Melt Analysis software (Bio-Rad). As shown in Fig. 1c there are three different clusters, one for each AA, TT and AT.

### Serum ACE1 and ACE2 quantification

Frozen (− 20 °C) serum samples of the hospitalized participants were thawed and the concentrations of ACE1 and ACE2 were measured according to manufacturer’s instructions, using the R&D-Systems duoset enzyme-linked immunosorbent assay (ELISA).

### Statistical analyses

The statistical analysis was performed with Graphpad Prism 9.

Due to the stringent requirements of the local ethics committee, we did not have any clinical data for the outpatient cohort, including sex and age. Thus, the genotype frequency of ACE polymorphisms in the whole cohort was compared with disease severity using the chi-square test. For the hospitalized cohort, we performed a logistic regression analysis adjusted for age and sex using the ID/AT genotype as a reference.

Data with normal distribution and equal variances from two groups were analyzed by using unpaired Student’s t-test. Mann–Whitney-test was used if data was not normally distributed. For comparing more than two groups, one-way analysis of variance (ANOVA) was performed and corrected for multiple comparisons with the Tukey test for normally distributed data. Kruskal Wallis test was applied for data that was not normally distributed and corrected with Dunn’s multiple comparisons test. Results are displayed as median with interquartile range. Two-sided p values < 0,05 were considered to be statistically significant. Correlation analysis was performed computing the Spearman’s rho (ρ). Receiver Operating Characteristic (ROC) analysis was done using the Wilson/Brown method for the confidence analysis.

## Results

### Participants

96 serum samples could be obtained for the outpatient group and were thus classified as ambulatory mild. A neutralization test (PRNT) confirmed the presence of neutralizing antibodies in all of them. Because of ethics committee regulations, no demographic data or information on comorbidities are available for these participants, except for a confirmed COVID-19 disease without hospitalization requirement. A total of 135 hospitalized patients from the four study centers (Berlin, Westerstede, Hamburg, Koblenz) were enrolled in the prospective clinical study PreBiSeCov [27]. As the clinical suspicion of COVID-19 was sufficient for inclusion in the study, 14 patients were ultimately found to have a diagnosis other than COVID-19, so they were excluded according to the study protocol. In addition, 9 patients withdrew consent or were incorrectly included by screening failure, e.g. due to immunosuppression. Furthermore, in 88 of these 112 participants in the PreBiSeCov study, the blood cakes could be analyzed and were therefore included in the study presented here. In all of them the presence of neutralizing antibodies to SARS-CoV-2 was confirmed. Of these 88 inpatients, 65.9% (n = 58) were male with a mean age of 60 years. 78 participants were moderately ill (64% male, median age 60 years), and 10 participants were severely ill or deceased (90% male, median age 71 years).

### Association of I/D and A/T polymorphisms with disease severity and inflammatory response

In the cohort of outpatients with mild disease severity, we found 21% II, 51% ID and 28% DD genotype carriers. The cohort of moderate and severe disease (inpatients) had a very similar distribution with 21% II, 48% ID and 31% DD genotype carriers. The distribution of the I/D and A/T polymorphism did not differ in the chi-square test between the three severity grades (p^I/D^ = 0,99; p^A/T^ = 0,339) (Table 3). Even in the hospitalized cohort we were not able to detect any differences in the logistic regression analysis.

**Table 3:** Distribution of I/D- and A/T polymorphisms regarding to COVID-19 disease severity

	Genotype	Severity				
		Mild (%)	Moderate (%)	Severe (%)	Xi^2	p
ACE I/D polymorphism	II	20 (20)	17 (22)	2 (20)	0.24	0.99
	ID	49 (51)	37 (47)	5 (50)		
	DD	27 (28)	24 (31)	3 (30)		
ACE A/T polymorphism	AA	33 (34)	22 (28)	5 (50)	4	0.39
	AT	48 (50)	42 (54)	4 (40)		
	TT	15 (16)	14 (18)	1 (10)		

Furthermore, our data showed no polymorphism-related difference in the age distribution (p^I/D^ = 0,97; p^A/T^ = 0,36), time to hospitalization (p^I/D^ = 0,93; p^A/T^ = 0,65) and time of hospitalization (p^I/D^ = 0,66; p^A/T^ = 0,16) for the inpatient group.

**Fig. 2:**
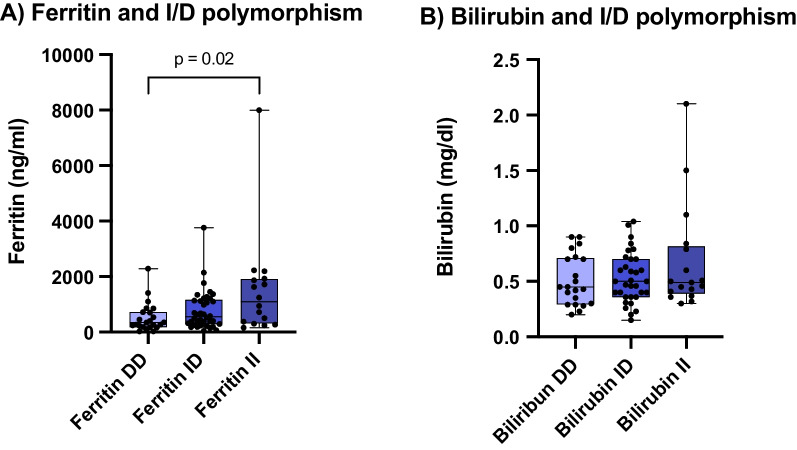
Initial serum ferritin levels and initial serum bilirubin levels linked to I/D polymorphisms, Kruskal–Wallis test. **A** Initial serum ferritin levels: n(DD) = 23 (left), n(ID) = 38 (mid), n(II) = 16 (right), *p = 0.02. **B** Initial serum bilirubin levels: n(DD) = 21 (left), n(ID) = 33 (mid), n(II) = 17 (right), *p = 0.58. Box and Whiskers: Median, lower and upper quartile, lower and upper extreme

**Fig. 3:**
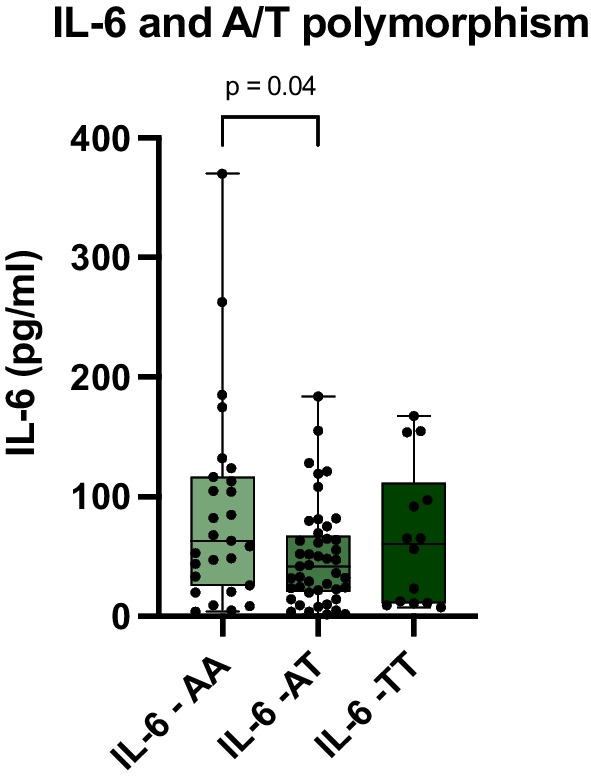
Initial Interleukin-6 (IL-6) levels linked to A/T polymorphism: Kruskal–Wallis test: n(AA) = 27 (left), n(AT) = 45 (mid), n(TT) = 14 (right), *p = 0.04. Box and Whiskers: Median, lower and upper quartile, lower and upper extreme

Clinical laboratory findings on admission in the moderate and severe groups (inpatients) showed no polymorphism-related differences in blood count, CRP, PCT and IL-6 for the I/D polymorphism. However, we found a significantly increased ferritin level in II compared to DD polymorphism (median(DD) = 342 ng/ml vs. median(II) = 1097 ng/ml, p^DD vs. II^ = 0.02) (Fig. 2), which was not associated with alterations in bilirubin or transaminases. With respect to the A/T polymorphism, there were significantly higher IL-6 levels in AA genotypes compared with AT genotypes (median(AT) = 41 pg/ml vs. median(AA) = 63 g/ml, p^AT vs. AA^ = 0.038) (Fig. 3) and a tendency towards increased leukocyte count in TT genotypes versus AA genotypes (median(TT) = 8,39 × 10^9^/l vs. median(aa) = 6,52 × 10^9^/l, p = 0.09), without any changes in the other previously mentioned parameters.

### Influence of RAAS on disease severity

**Fig. 4:**
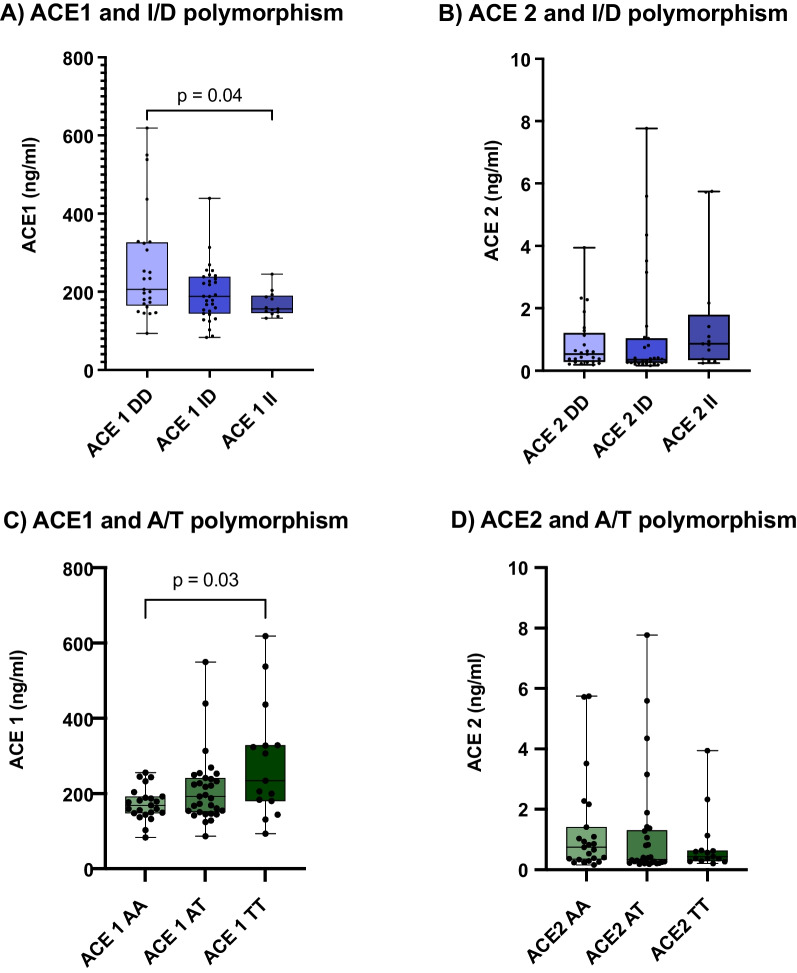
Serum Angiotensin-converting enzyme (ACE) 1 and 2 levels linked to the I/D and A/T polymorphisms, Kruskal–Wallis test. **A** ACE1 and I/D polymorphism: n(DD) = 25 (left), n(ID) = 31 (mid), n(II) = 13 (right), *p = 0.04. **B** ACE2 and I/D polymorphism: n(DD) = 25 (left), n(ID) = 31 (mid), n(II) = 13 (right), *p = 0.13.**C** ACE1 and A/T polymorphism: n(AA) = 23 (left), n(AT) = 31 (mid), n(TT) = 15 (right), *p = 0.03. **D** ACE2 and A/T polymorphism: n(AA) = 23 (left), n(AT) = 31 (mid), n(TT) = 15 (right), *p = 0.30. Box and Whiskers: Median, lower and upper quartile, lower and upper extreme

To assess the influence of the ACE-polymorphism on the RAAS, we quantified ACE1 and ACE2 serum concentrations in the sera of the cohort subjects. ACE1 serum levels were significantly increased in the DD compared to the II genotypes (median(DD) = 206 ng/ml vs. median(II) = 156 ng/ml, p^DD vs.II^ = 0.0426) and in the TT compared to the AA genotypes (median(AA) = 168 ng/ml, median(TT) = 233 ng/ml, p^TTvs.AA^ = 0.0341). As expected, the A/T and I/D polymorphisms did not affect ACE2 levels in this cohort (Fig. 4).

To determine the influence of ACE1 and ACE2 on COVID-19 disease course, we analyzed the correlation of serum ACE1 and ACE2 concentrations to disease severity, clinical course and gender. In our cohort, only one participant died. To facilitate statistical analysis, this participant was pooled with the severely ill group. The clinical laboratory showed significantly lower lymphocyte counts, significantly higher CRP, PCT and IL-6 levels and no significant difference in the ferritin levels in the cohort with severe disease compared to the moderate disease cohort. There was no significant difference in ACE1 levels between moderately ill and severely ill (medianACE1(moderate) = 188 ng/ml vs. medianACE1(severe) = 159 ng/ml, p^ACE1^ = 0.23). However, between these groups, there was a trend toward lower ACE2 levels in the severe cohort (medianACE2(moderate) = 0,6 ng/ml vs. medianACE2(severe) = 0,37 ng/ml, p^ACE2^ = 0.08).

**Fig. 5:**
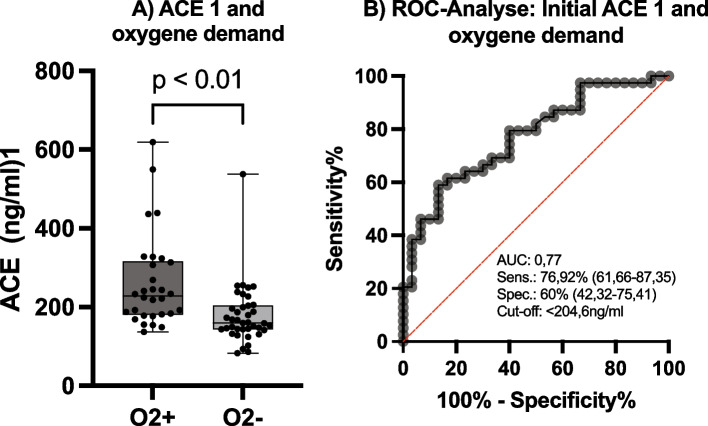
Initial Angiotensin-converting enzyme (ACE) 1 serum level in patients and oxygene demand and ROC-analyse. **A** Patients with oxygen demand (left) and without oxygen demand (right) (A); n = 69, *p < 0.01; Box and Whiskers: Median, lower and upper quartile, lower and upper extreme. **B** Receiver operating characteristic (ROC) curve analysis for initial ACE 1 level regarding oxygen demand, cut-off level < 204.6 ng/ml; AUC: 0.77, p < 0.01

Most importantly, patients who required supplemental oxygen had significantly elevated ACE-1 levels compared with participants who did not require oxygen (median(ACE1 O_2_^+^) = 228 ng/ml, median(ACE 1, O_2_^−^) = 159 ng/ml, p = 0.0001*) with no difference in ACE2 levels (median(ACE2 O_2_^+^) = 0.47 ng/ml; median(ACE2 O_2_^−^) = 0.40 ng/ml, p = 0.46). ROC curve analysis showed serum ACE1 to be a potential diagnostic biomarker for the need for oxygen supplementation, with an area under the ROC curve of 0.77. The sensitivity, specificity, and resulting cutoff values are shown in Fig. 5.

**Fig. 6:**
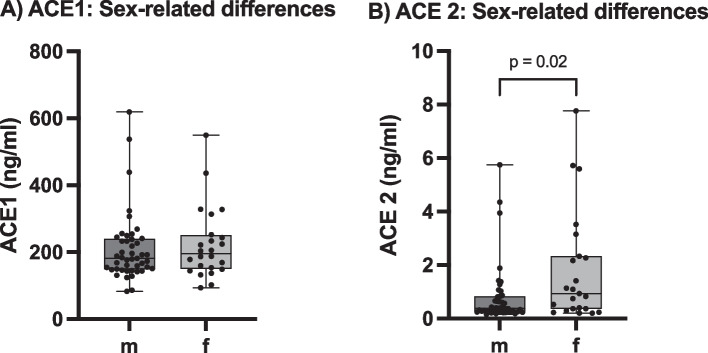
Sex-related differences in serum ACE1 and ACE2 levels, *m:* male, *f:* female; Mann–Whitney test. **A** Serum ACE1 levels, n = 69, p = 0.58; **B** Serum ACE2 levels, n = 68, *p = 0.02. Box and Whiskers: Median, lower and upper quartile, lower and upper extreme

Analysis of the influence of gender on the RAAS revealed no significant differences in ACE1 levels (median(ACE1 m) = 181.7 ng/ml, median(ACE1 f) = 195.7 ng/ml, p = 0.58). Nevertheless, females had significantly higher ACE2 levels with a median level of 0.93 ng/ml compared to males with a median level of 0.37 ng/ml (p = 0.023) (Fig. 6).

## Discussion

The present study aims first to investigate the influence of I/D and A240T ACE1 gene polymorphisms on the severity and inflammation of COVID-19 disease, combined with an assessment of the impact of these polymorphisms on serum ACE1 and ACE2 levels. Second, an analysis of ACE1 and ACE2 serum levels in COVID-19 patients was performed in relation to clinical course and routine laboratory parameters.

Since the beginning of the COVID-19 pandemic, the influence of genetic predisposition on the severity of the disease has been the subject of numerous studies. In addition to studies on blood groups [29] and the kallikrein system [30], there is a large number of studies on the renin-angiotensin system, as the ACE2 receptor plays a crucial role in cellular invasion [31–33]. Nevertheless, our data showed no polymorphism differences of the ACE1 gene in outpatients compared with inpatients or in the severity of COVID-19. Our findings on the I/D polymorphism might be controversial to a certain extent to Gomez et al. who revealed an unfavorable course for the DD genotype in a Spanish cohort, attributed to an association with hypertension [34]. About 50% (n = 46) of our inpatients had known arterial hypertension and were receiving antihypertensive therapy. In the overall view of our central European, German cohort and in the sub-cohort of patients with arterial hypertension, we did not see an unfavorable course for the DD genotype as Gomez et al. did. Rather, our results are consistent with the findings of Mohlendick et al. [35] who was also unable to confirm the results of Gomez et al. There are regionally different risk-association for the ACE D allele that were known before the emergence of COVID-19. The D allele is a known independent risk factor for pneumonia in the elderly Asian individuals in contrast to the elderly Dutch Caucasians [36, 37]. Thus, the regionally varying risks associated with the D allele in relation to COVID-19 are warranted further investigation.

In addition to the importance of the ACE2 receptor for cellular uptake of SARS-CoV2, the RAAS has a pivotal role in pro-inflammatory and anti-inflammatory regulatory pathways. Indeed, we found higher levels of ferritin in the DD compared to the II polymorphism. We consider ferritin primary as acute phase protein in our patients, as other hepatological parameters were normal. As a highly sensitive protein of the acute phase reaction, ferritin counteracts the consequences of pro-inflammatory reaction and the associated oxidative stress. In the serum of our inpatients at admission, ferritin was significantly lower in DD compared to II genotype. Importantly, concentrations of ACE1 in serum were significantly higher in DD compared to II genotype. This association between the D allele and higher ACE1 concentrations is well-known [38]. As described above, ACE1 has a proinflammatory effect via the AT1 receptor. Although we could not find any differences in the distribution of disease severity (according to the WHO-criteria) regarding the I/D polymorphisms and ACE1 levels, ACE1 was significantly increased in the group of study participants who were dependent on oxygen supplementation. This is in line with the findings of Guler et al., who was also not able to detect differences in the ACE activity regarding the disease severity [39]. Therefore, an elevated ACE1 level as a diagnostic biomarker could predict the need for oxygen supply with an area under the ROC curve of 0.77 (Fig. 5, right). Although the D allele is not associated with disease severity in this study cohort, it is associated with an unfavorable inflammatory response.

In contrast, ACE2 modulates the RAAS via the Mas receptor towards an anti-inflammatory response [40]. Therefore, it seems consistent that in our cohort severely affected patients tended to have lower ACE2 levels. Both findings fit an altered inflammatory response depending on the I/D polymorphism or the RAAS. According to Gemmati et al., a dysbalanced RAAS, particularly an imbalance of ACE1/ACE2, could lead to higher expression of inflammatory mediators/receptors in both sexes. They hypothesize that males with COVID-19 may have a worse clinical course due to the mosaic advantage of female X heterozygosity [19].

In addition to the trend towards increased ACE2 levels in the moderately ill cohort, we report significantly higher ACE2 levels in women compared to men. Despite similar SARS-CoV-2 infection rates between the sexes, men account for the greater proportion of deaths in most countries of the world [41]. It is therefore plausible that ACE2 and its genetic variants also have sex-specific effects despite possible differences in comorbidities. Thus, despite the relatively small sample size of our cohorts, our results are consistent with the hypothesis of the aforementioned study.

The A/T polymorphism is also involved in the inflammatory response in our cohorts. Elevated ACE1 levels could be detected in the serum of patients with the TT polymorphism, for example, who has significantly increased ACE1 levels compared to the AA polymorphism. To our knowledge, we are the first to link increased ACE1 levels with TT polymorphism in COVID-19 patients. Furthermore, we see a tendency towards increased leukocyte count (p = 0.09) in TT versus AA genotypes and significantly increased IL-6 levels in the cohort with AA versus AT genotypes. The trend towards increased leukocyte count reflects ACE1 levels in the same genotype, but requires further investigation.

The findings of this study are in line with those of Tepasse et al., who demonstrated a rise in DBK1-5 fragment of labelled bradykin (DBK) and consequent excessive ACE activity, with insufficient counter-regulation of ACE2 in severe cases. In this study a strong correlation between DBK1-5 formation and the mortality parameter SAPS II, among other factors was demonstrated [42].

This study demonstrates the impact of Sars-CoV II infection on a fine-tuned ACE1 ACE2 balance. SARS CoV II reduces ACE2 activity and expression. This in turn leads to an increase in the Ang-2/AT-1 signaling pathway and thus to a shift in the pro-inflammatory effect of the RAS system. In individuals who already express less ACE2, such as men, the reduction in ACE2 activity by SARS viruses might trigger a cascade of deleterious effects through an increased imbalance between the effects of the products of ACE1 and ACE2. Especially in patients with genetically elevated ACE1 levels, this constellation may be unfavorable.

### Strengths and limitations

An important limitation is the retrospective design of our study and the absence of a SARS-CoV-2 negative control group. Consequently, demographic data of the outpatient cohort are missing, as required by the ethics committee. All these factors are sources of bias. Another limitation of our study was the limited number of patients enrolled in the investigation, contributing to reduced statistical significance and power. Our study was not designed to investigate the cellular mechanisms underlying elevated serum ACE2 levels. Whether this is pathognomonic for SARS-CoVII disease, a concomitant disease, previous illness, sex, or interindividual differences cannot be conclusively investigated in this study and requires further research.

Mortality in our cohort was very low. However, the distribution of disease severity, age and pre-existing conditions of the inpatient cohort was in line with expectations at the beginning of the pandemic for secondary and tertiary care centers in Germany and can therefore be considered representative for them.

## Conclusion

Taken together, despite not recording any differences in disease severity between the I/D and the A/T polymorphism in terms of the severity of COVID-19 infection, we were able to demonstrate an increased pro-inflammatory regulation of the RAAS in the patients with oxygen demand and elevated serum ACE2 levels in women, probably indicating an increased anti-inflammatory immune response.

## Data Availability

Data and materials are available on request, subject to data protection agreements.

## Abbreviations

ACE: Angiotensin converting enzyme
ACEPolyCovBw: Analysis of ACE and ACE II polymorphism in patients with SARS-CoV-2 infection Bundeswehr
ARDS: Acute respiratory distress syndrome
COVID-19: Coronavirus disease 2019
PreBiSeCov: Predictive biomarkers for the severe course of COVID-19
PRNT: Plaque reduction neutralization test
RT: Room temperature
SARS-CoV-2: Severe acute respiratory syndrome coronavirus 2

## References

[1] Huang C, Wang Y, Li X, Ren L, Zhao J, Hu Y (2020). Clinical features of patients infected with 2019 novel coronavirus in Wuhan, China. Lancet.

[2] Hoffmann M, Kleine-Weber H, Schroeder S, Kruger N, Herrler T, Erichsen S (2020). SARS-CoV-2 cell entry depends on ACE2 and TMPRSS2 and is blocked by a clinically proven protease inhibitor. Cell.

[3] Li MY, Li L, Zhang Y, Wang XS (2020). Expression of the SARS-CoV-2 cell receptor gene ACE2 in a wide variety of human tissues. Infect Dis Poverty.

[4] Zou X, Chen K, Zou J, Han P, Hao J, Han Z (2020). Single-cell RNA-seq data analysis on the receptor ACE2 expression reveals the potential risk of different human organs vulnerable to 2019-nCoV infection. Front Med.

[5] Hamming I, Cooper ME, Haagmans BL, Hooper NM, Korstanje R, Osterhaus AD (2007). The emerging role of ACE2 in physiology and disease. J Pathol.

[6] Hamming I, Timens W, Bulthuis ML, Lely AT, Navis G, van Goor H (2004). Tissue distribution of ACE2 protein, the functional receptor for SARS coronavirus. A first step in understanding SARS pathogenesis. J Pathol.

[7] Narula S, Yusuf S, Chong M, Ramasundarahettige C, Rangarajan S, Bangdiwala SI (2020). Plasma ACE2 and risk of death or cardiometabolic diseases: a case-cohort analysis. Lancet.

[8] Pradhan A, Olsson PE (2020). Sex differences in severity and mortality from COVID-19: are males more vulnerable?. Biol Sex Differ.

[9] Zhang J, Wang X, Jia X, Li J, Hu K, Chen G (2020). Risk factors for disease severity, unimprovement, and mortality in COVID-19 patients in Wuhan, China. Clin Microbiol Infect.

[10] Warner FJ, Lew RA, Smith AI, Lambert DW, Hooper NM, Turner AJ (2005). Angiotensin-converting enzyme 2 (ACE2), but not ACE, is preferentially localized to the apical surface of polarized kidney cells. J Biol Chem.

[11] Santos RA, Ferreira AJ, Simoes ESAC (2008). Recent advances in the angiotensin-converting enzyme 2-angiotensin(1–7)-Mas axis. Exp Physiol.

[12] Clarke NE, Turner AJ (2012). Angiotensin-converting enzyme 2: the first decade. Int J Hypertens.

[13] Li XC, Zhang J, Zhuo JL (2017). The vasoprotective axes of the renin-angiotensin system: Physiological relevance and therapeutic implications in cardiovascular, hypertensive and kidney diseases. Pharmacol Res.

[14] Li W, Moore MJ, Vasilieva N, Sui J, Wong SK, Berne MA (2003). Angiotensin-converting enzyme 2 is a functional receptor for the SARS coronavirus. Nature.

[15] Tomita H, Ina Y, Sugiura Y, Sato S, Kawaguchi H, Morishita M (1997). Polymorphism in the angiotensin-converting enzyme (ACE) gene and sarcoidosis. Am J Respir Crit Care Med.

[16] Specks U, Martin WJ, Rohrbach MS (1990). Bronchoalveolar lavage fluid angiotensin-converting enzyme in interstitial lung diseases. Am Rev Respir Dis.

[17] Orte C, Polak JM, Haworth SG, Yacoub MH, Morrell NW (2000). Expression of pulmonary vascular angiotensin-converting enzyme in primary and secondary plexiform pulmonary hypertension. J Pathol.

[18] Verma S, Abbas M, Verma S, Khan FH, Raza ST, Siddiqi Z (2021). Impact of I/D polymorphism of angiotensin-converting enzyme 1 (ACE1) gene on the severity of COVID-19 patients. Infect Genet Evol.

[19] Gemmati D, Bramanti B, Serino ML, Secchiero P, Zauli G, Tisato V (2020). COVID-19 and individual genetic susceptibility/receptivity: role of ACE1/ACE2 genes, immunity, inflammation and coagulation. Might the double X-chromosome in females be protective against SARS-CoV-2 compared to the single X-chromosome in males?. Int J Mol Sci.

[20] Suryamohan K, Diwanji D, Stawiski EW, Gupta R, Miersch S, Liu J (2021). Human ACE2 receptor polymorphisms and altered susceptibility to SARS-CoV-2. Commun Biol.

[21] Delanghe JR, Speeckaert MM, De Buyzere ML (2020). COVID-19 infections are also affected by human ACE1 D/I polymorphism. Clin Chem Lab Med.

[22] Zhu X, Bouzekri N, Southam L, Cooper RS, Adeyemo A, McKenzie CA (2001). Linkage and association analysis of angiotensin I-converting enzyme (ACE)-gene polymorphisms with ACE concentration and blood pressure. Am J Hum Genet.

[23] Firouzabadi N, Tajik N, Shafiei M, Ebrahimi SA, Bakhshandeh H (2011). Interaction of A-240T and A2350G related genotypes of angiotensin-converting enzyme (ACE) is associated with decreased serum ACE activity and blood pressure in a healthy Iranian population. Eur J Pharmacol.

[24] Villard E, Tiret L, Visvikis S, Rakotovao R, Cambien F, Soubrier F (1996). Identification of new polymorphisms of the angiotensin I-converting enzyme (ACE) gene, and study of their relationship to plasma ACE levels by two-QTL segregation-linkage analysis. Am J Hum Genet.

[25] da Agostini L, Cunha WR, Silva NNT, Melo AS, Moreira LB, Almeida TC (2023). Angiotensin-converting enzyme gene (ACE) polymorphisms are associated with dysregulation of biochemical parameters in hypertensive patients. Mol Biol Rep.

[26] Asselta R, Paraboschi EM, Mantovani A, Duga S (2020). ACE2 and TMPRSS2 variants and expression as candidates to sex and country differences in COVID-19 severity in Italy. Aging (Albany NY).

[27] Zobel CM, Wenzel W, Kruger JP, Baumgarten U, Wagelohner T, Neumann N (2023). Serum interleukin-6, procalcitonin, and C-reactive protein at hospital admission can identify patients at low risk for severe COVID-19 progression. Front Microbiol.

[28] Characterisation WHOWGotC, Management of C-i. A minimal common outcome measure set for COVID-19 clinical research. Lancet Infect Dis. 2020;20(8):e192-e7. 10.1016/S1473-3099(20)30483-7 PMC7292605 32539990

[29] Zhao J, Yang Y, Huang H, Li D, Gu D, Lu X (2021). Relationship between the ABO blood group and the coronavirus disease 2019 (COVID-19) susceptibility. Clin Infect Dis.

[30] Martens CP, Van Mol P, Wauters J, Wauters E, Gangnus T, Noppen B (2022). Dysregulation of the kallikrein-kinin system in bronchoalveolar lavage fluid of patients with severe COVID-19. EBioMedicine.

[31] Bekassy Z, Lopatko Fagerstrom I, Bader M, Karpman D (2022). Crosstalk between the renin-angiotensin, complement and kallikrein-kinin systems in inflammation. Nat Rev Immunol.

[32] Carvalho PR, Sirois P, Fernandes PD (2021). The role of kallikrein-kinin and renin-angiotensin systems in COVID-19 infection. Peptides.

[33] Shibeeb S, Khan A (2022). ABO blood group association and COVID-19. COVID-19 susceptibility and severity: a review. Hematol Transfus Cell Ther..

[34] Gomez J, Albaiceta GM, Garcia-Clemente M, Lopez-Larrea C, Amado-Rodriguez L, Lopez-Alonso I (2020). Angiotensin-converting enzymes (ACE, ACE2) gene variants and COVID-19 outcome. Gene.

[35] Mohlendick B, Schonfelder K, Breuckmann K, Elsner C, Babel N, Balfanz P (2021). ACE2 polymorphism and susceptibility for SARS-CoV-2 infection and severity of COVID-19. Pharmacogenet Genom.

[36] Morimoto S, Okaishi K, Onishi M, Katsuya T, Yang J, Okuro M (2002). Deletion allele of the angiotensin-converting enzyme gene as a risk factor for pneumonia in elderly patients. Am J Med.

[37] van de Garde EM, Endeman H, Deneer VH, Biesma DH, Sayed-Tabatabaei FA, Ruven HJ (2008). Angiotensin-converting enzyme insertion/deletion polymorphism and risk and outcome of pneumonia. Chest.

[38] Pontremoli R, Sofia A, Tirotta A, Ravera M, Nicolella C, Viazzi F (1996). The deletion polymorphism of the angiotensin I-converting enzyme gene is associated with target organ damage in essential hypertension. J Am Soc Nephrol.

[39] Avanoglu Guler A, Tombul N, Aysert Yildiz P, Ozger HS, Hizel K, Gulbahar O (2021). The assessment of serum ACE activity in COVID-19 and its association with clinical features and severity of the disease. Scand J Clin Lab Investig.

[40] Beyerstedt S, Casaro EB, Rangel EB (2021). COVID-19: angiotensin-converting enzyme 2 (ACE2) expression and tissue susceptibility to SARS-CoV-2 infection. Eur J Clin Microbiol Infect Dis.

[41] Pijls BG, Jolani S, Atherley A, Derckx RT, Dijkstra JIR, Franssen GHL (2021). Demographic risk factors for COVID-19 infection, severity, ICU admission and death: a meta-analysis of 59 studies. BMJ Open.

[42] Tepasse PR, Vollenberg R, Steinebrey N, Konig S (2022). High angiotensin-converting enzyme and low carboxypeptidase N serum activity correlate with disease severity in COVID-19 patients. J Pers Med..

